# Identification and characterization of the first pectin methylesterase gene discovered in the root lesion nematode *Pratylenchus penetrans*

**DOI:** 10.1371/journal.pone.0212540

**Published:** 2019-02-22

**Authors:** Cláudia S. L. Vicente, Lev G. Nemchinov, Manuel Mota, Jonathan D. Eisenback, Kathryn Kamo, Paulo Vieira

**Affiliations:** 1 ICAAM - Instituto de Ciências Agrárias e Ambientais Mediterrânicas, Instituto de Investigação e Formação Avançada, Universidade de Évora, Pólo da Mitra, Évora, Portugal; 2 Molecular Plant Pathology Laboratory, Agricultural Research Service, United States Department of Agriculture, Beltsville, Maryland, United States of America; 3 Departamento de Biologia & ICAAM - Instituto de Ciências Agrárias e Ambientais Mediterrânicas, Universidade de Évora, Pólo da Mitra, Évora, Portugal; 4 School of Plant Environmental Science, Virginia Tech, Blacksburg, Virginia, United States of America; 5 Floral and Nursery Plants Research Unit, United States of National Arboretum, United States Department of Agriculture, Beltsville, Maryland, United States of America; Institute of Natural Resources and Agrobiology of Salamanca (IRNASA-CSIC), SPAIN

## Abstract

Similar to other plant-parasitic nematodes, root lesion nematodes possess an array of enzymes that are involved in the degradation of the plant cell wall. Here we report the identification of a gene encoding a cell wall-degrading enzyme, pectin methylesterase PME (EC 3.1.1.11), in the root lesion nematode *Pratylenchus penetrans*. Both genomic and coding sequences of the gene were cloned for this species, that included the presence of four introns which eliminated a possible contamination from bacteria. Expression of the *Pp-pme* gene was localized in the esophageal glands of *P*. *penetrans* as determined by *in situ* hybridization. Temporal expression of *Pp-pme in planta* was validated at early time points of infection. The possible function and activity of the gene were assessed by transient expression of *Pp-pme* in plants of *Nicotiana benthamiana* plants via a Potato virus X-based vector. To our knowledge, this is the first report on identification and characterization of a PME gene within the phylum Nematoda.

## Introduction

The plant cell wall plays an important role in various fundamental physiological processes of plant growth and development, such as maintaining the integrity of cellular content, morphogenesis, and cell signaling. In addition, the cell wall is the primary interface for most plant-pathogen interactions, since it is the first physical barrier against invasion and infection [1, 2]. The primary cell wall has an intricate structure composed of a complex network of cellulose microfibrils interconnected within a matrix of polysaccharides, including pectins, hemicelluloses, and glycoproteins [3, 4]. Pectin, a highly abundant polysaccharide, is an important component of both primary and secondary cell walls, forming the main component of the middle lamella [5, 6]. Modification of the pectin network is tightly regulated by the action of pectinolytic enzymes and pectinases (e.g. pectate lyases, pectin methylesterases and polygalacturonases), whereas the cellulose/hemicellulose network is targeted by cellulolytic enzymes (e.g. endo- and exoglucanases) and hemicellulases [7, 8].

Pectin methylesterases (PMEs; EC 3.1.1.11) are a group of enzymes belonging to the carbohydrate esterase family 8 (CE8). They catalyze hydrolysis of the methyl ester of homogalacturonan, the backbone of pectin, which releases acidic pectins and methanol that facilitates the modification of the plant cell wall and its subsequent degradation [9, 10]. Pectin, de-esterified by PMEs, becomes more susceptible to degradation by other pectinases (e.g. polygalacturonase, pectate lyase and rhamnogalacturonan lyase), which alters the texture and integrity of the cell wall and contributes to its loosening [11].

PMEs are widely present in plants, which encode a large number of isoforms that play important roles in plant development and major physiological processes [9], such as microsporogenesis, pollen growth, seed germination, root development, polarity of leaf growth, stem elongation, fruit ripening, loss of tissue integrity, cell wall extension, and softening [9, 12–15]. Moreover, PMEs have also been reported to play an important role in response to fungal [15] and bacterial pathogens, and are required for the systemic spread of the tobacco mosaic virus in plants [16].

Plant pathogenic microorganisms (e.g. bacteria and fungi) are very efficient in degrading plant cell wall polysaccharides using their own battery of cell wall-degrading enzymes (CWDEs). These CWDEs are normally secreted into the host tissues and efficiently degrade plant cells, allowing pathogens access to the cells, or in some cases, to utilize these polysaccharides as a source of nutrients for their own growth and development. Among these are PMEs, which play key roles in the infection process of plant pathogens by breaking down of the plant cell wall, which is a primary requirement to successful invasion of a host plant. Significant differences between PMEs of plants and microorganisms have been found [9]. For example, fungal PMEs appear to have a broader range of adaptability to substrates [17]. Secreted PMEs of bacteria and fungi are involved in invasion of the host plant and pathogenicity. The breakdown of pectin by these PMEs can lead to the maceration and soft-rotting of plant tissues which is a characteristic phenotype of soft-rot diseases [18, 19].

Although the synthesis of PMEs has been often attributed to the free-living or endosymbiotic organisms that inhabit the gut of some insects, several studies confirmed that these phytophagous insects are also able to encode PMEs through their own endogenous genes [20–22]. In this context, PME encoding genes have been identified for a few species belonging to the family Curculionidae (weevils and bark beetles) [20, 23]. With the increase of genome and transcriptome datasets available for different animal species, additional PME-encoding genes have been recently identified for the whitefly *Bemisia tabaci* Gennadius, 1889 (Insecta: Hemiptera: Aleyrodidae) and the springtail soil arthropod *Folsomia candida* Willem, 1902 (Entognatha: Collembola) [24, 25]. Although functional analyses are still lacking for most of the animal PMEs identified, emerging data indicate that PME activity is important during insect-plant interactions. The PMEs of the rice weevil (*Sitphilus oryzae* L., 1763) were found to act synergistically with other pectinases to enable the breakdown of the complex polysaccharide pectin network that allows access of the cell contents [22, 26, 27]. Other than these reports, PME encoding genes have not yet been identified in other animals so far.

Root lesion nematodes (RLNs) are migratory, endoparasitic nematodes that are able to parasitize a broad host range and cause extensive root damage to plant hosts [28]. All motile stages of RLN can penetrate the roots, which feed predominately on the root cortical tissues and causes the formation of lesions, browning, and cell death [28]. The successful invasion of roots by RLNs is related to their ability to overcome the barrier imposed by the plant cell wall. Like other plant-parasitic nematodes (PPNs), RLNs are equipped with a protrusible stylet that mechanically disrupts the cell wall and through which CWDEs are secreted to facilitate penetration and migration of the nematode through host roots.

*Pratylenchus penetrans* (Cobb, 1917) Filipjev and Schuurmans Stekhoven, 1941 is regarded as one of the most destructive species of this genus because of its ability to parasitize a wide range of economically important host plants (e.g. alfalfa, corn, and potato), and its broad geographic distribution [29]. The core set of genes encoding CWDEs identified for *P*. *penetrans* so far comprises β-1,4-endoglucanases (GH5), pectate lyases (PL3), arabinogalactan endo-1,4-β-galactosidases (GH53), xylanases (GH30), and expansin-like genes [30]. As in other PPNs, the majority of these CWDEs are localized in the esophageal gland cells of *P*. *penetrans*, and are actively produced during the early time points of plant infection [31]. Interestingly, a refined data mining of the *P*. *penetrans* transcriptome [30] resulted in the identification of a transcript encoding a PME gene, here after named as *Pp-pme*. Here we report the identification and molecular characterization of this *Pp-pme* gene, which is, to the best of our knowledge, the first report of a PME encoding gene within the phylum Nematoda.

## Material and methods

### Nematode collection and extraction

Two different isolates of *P*. *penetrans* were used in this study: 1) NL 10p RH collected in Beltsville, MD, USA, and 2) A44L4 from potato (*Solanum tuberosum* L.) fields in Coimbra, Portugal. Both isolates were maintained and multiplied *in vitro* in roots of corn (*Zea mays* L. cv. ‘Iochief’) growing in Murashige and Skoog basal medium containing vitamins (MS) (Sigma-Aldrich, MO, USA), 3% (w/v) of sucrose (pH 5.8) and solidified with 1.5% (w/v) agar (Sigma-Aldrich, MO, USA). Nematodes were re-cultured every two months into new roots of corn and maintained in the dark at 28°C. Nematodes were extracted from infected corn roots as described in Vieira et al. [30].

### Isolation of *Pp-pme* genomic DNA and cDNA sequences

During our previous analyses of the transcriptome assembly generated for *P*. *penetrans* [30], a partial transcript of 415 bp showing similarity to a bacterial pectin methylesterase gene (WP_090106049.1) was identified. In order to obtain the corresponding full-length coding sequence, BLAST searches were initially performed against the skimming genome assembly of *P*. *penetrans* [32]. Based on the assembly of the retrieved contigs, primers were designed to flank the *in silico* predicted fragment (S1 Table), as well as the putative full-length protein-encoding *Pp-pme* sequence, including partial sequences of both 5´and 3´regions (S1 Table). DNA extraction was performed from mixed stages (eggs, juveniles, females and males) of *P*. *penetrans* using the PureLink Genomic DNA Mini kit (Invitrogen, Carlsbad, CA, USA), following the manufacturer’s instructions. Total RNA was extracted from mixed life stages of *P*. *penetrans* using the RNeasy Plant Mini Kit (Qiagen, Valencia, CA, USA) according to the manufacturer’s instructions. RNA was then treated with RNase-Free DNase (Qiagen, Valencia, CA, USA) before reverse transcription. The quantity and quality of the extracted RNA was assessed with a ND-1000 Nanodrop spectrophotometer (NanoDrop). The first strand cDNA was synthesized using the iScript first-strand synthesis kit (Bio-Rad, Hercules, CA, USA) following the manufacturer’s instructions. The putative *Pp-pme* genomic coding region of the isolate NL 10p RH was obtained by PCR amplification using 5 μl of the nematode DNA extract, 1x PCR buffer, 1 U Platinum Taq DNA polymerase (Invitrogen) and 0.2 μM of each primer in a 50 μl solution. A PTC-200 Peltier thermocycler was used for amplification using the following PCR conditions: one denaturation step at 94°C for 2 min, followed by 30 cycles at 94°C for 30 secs, 55°C for 30 secs, 72°C for 3 min, and a final extension step of 10 min at 72°C. For amplification of the cDNA sequences of NL 10p RH and A44L4 isolates, 1 μl of cDNA was used which derived from each of the corresponding mixed-stage libraries, followed by similar PCR conditions described above, with an adjusted extended time (i.e. 1 minute per kb was used). The corresponding genomic and cDNA amplicons of *P*. *penetrans* were separated on a 1% agarose gel and stained with SYBRSAFE, and the corresponding bands were recovered from the gel using the MinElute Gel Extraction kit (Qiagen, Valencia, CA, USA). Both amplicons were then ligated to the TOPO TA Cloning kit (Invitrogen, Carlsbad, CA, USA) using the protocol provided by the manufacturer, and transformed into *E*. *coli* TOP10 competent cells (Invitrogen, Carlsbad, CA, USA). PCR colony positive clones were grown in 3 mL of LB overnight at 37°C followed by plasmid DNA extraction (QIAprep Spin Miniprep kit, Qiagen, Valencia, CA, USA). DNA and cDNA clones were verified by automated sequencing using the universal M13 primers by Macrogen USA (MD, USA). Unless otherwise stated, all the following analyses were conducted with *P*. *penetrans* isolate NL 10p RH.

### Sequence analysis

To identify introns in the genomic sequence, both cDNA and genomic sequences were aligned using MUSCLE [33]. Gene schematics were generated with the Exon–Intron Graphic maker available at WormWeb.org. The nucleotide and translated amino acid sequences were analyzed for similarity to other genes and proteins using BLAST analyses against the NCBI non-redundant nucleotide and protein databases (http://www.ncbi.nlm.nih.gov/), the nematode.net (http://www.nematode.net/), and NEMBASE4 database (http://www.nematodes.org/nembase4/). In addition, protein sequence analyses were conducted using the following programs: SignalP 4.0 for prediction of protein signal peptide [34]; ProteParam for determination of the protein molecular mass and theoretical isoelectric point [35]; and CLC Main Workbench v. 8.0 software for protein secondary structure predictions. InterPro scan was performed using the public available software package (https://www.ebi.ac.uk/interpro/sequencesearch/iprscan). The overall GC content (%) and codon usage (GC1, GC2 and GC3 corresponding to the 1^st^, 2^nd^ and 3^rd^ position in the codon triplet, respectively) were calculated using the on-line EMBOSS cusp package (http://www.bioinformatics.nl/cgi-bin/emboss/cusp). Modelling of the tertiary structure of the *P*. *penetrans* PME and the corresponding PME TOP hits of Bacteria/ Fungi/Plants/Insects were performed using Phyre2 in the normal mode analysis [36] (http://www.sbg.bio.ic.ac.uk/phyre2). Structural classification of the translated protein followed the convention of SCOPe (Structural Classification of Proteins—extended, http://scop.berkeley.edu/) [37].

BLASTp search was done against the non-redundant (nr) protein database at NCBI, and sequence selection was based on the TOP hits of Bacteria/Archaea/Fungi/Plants/Insects, excluding sequences of the same species with 100% identity. The percentage of protein identity (identical residues in alignment positions) between the *P*. *penetrans* PME and those of the other organisms was determined by pairwise comparison. Multiple sequence alignment of PME was conducted with MAFFT algorithm [38] with default settings, trimmed with TRIMAL [39] and manually curated and edited for the selection of protein domain. The phylogenetic relationships were estimated using maximum likelihood (ML) analysis. The best model for protein evolution, determined with AMINOSAN [40] was the “Whelan and Goldman” (WAG) model with discrete gamma distribution (number of gamma categories = 8). The robustness of ML analysis was inferred using 1,000 bootstrap replicates.

### *Pp-pme* expression at different nematode developmental stages

RNA of different nematode developmental stages [eggs, juveniles (J2 to J4), males, and females] was obtained from 150 nematodes and then isolated using the same kit and conditions mentioned above. Expression of the *Pp-pme* at different developmental stages of *P*. *penetrans* was analyzed by semi-quantitative RT-PCR using a pair of primers that amplify a *Pp-pme* fragment of 221 bp (S1 Table). The *P*. *penetrans 18S* rDNA gene was used as the reference gene (S1 Table). PCR products were separated on a 1% agarose gel and stained with SYBR Safe.

### *In situ* hybridization of *Pp-pme* transcripts

To assess the localization of *Pp-pme* transcripts, whole mount *in situ* hybridization was performed using all stages of *P*. *penetrans* NL 10p RH following the protocol of de Boer et al. [41]. For localization of the *Pp-pme* transcripts, the same primers described above for RT-PCR analyses were used, while a fragment of 241 bp of the *Pp-eng-1* gene was used as positive control in this experiment (S1 Table). The resulting PCR products were used as a template for generation of sense and antisense DIG-labeled *Pp-pme* probes using a DIG-nucleotide labeling kit (Roche, Indianapolis, IN, USA). Hybridized probes within the nematode tissues were detected using anti-DIG antibody conjugated to alkaline phosphatase and its substrate. Nematode sections were then observed using a Nikon Eclipse 5*i* light microscope.

### *Pp-pme* transcript expression in host roots after nematode infection

To validate the expression of *Pp-pme* during its interaction with plants, *in vitro* assays were conducted using roots of alfalfa, corn, potato, and soybean and challenged with *P*. *penetrans* as described by Vieira et al. [42]. Total RNA was then extracted from a pool of infected roots at 1, 3, 7 or 11 days after nematode infection (DAI) as mentioned previously. Semi-quantitative RT-PCR analyses were performed as described above. Reference genes included an *actin* gene for corn and potato, the *Ubiquitin-3* (Ubi3) for soybean, and the *NP_001237047* gene for alfalfa (S1 Table).

### Expression of *Pp-pme in planta*

For expression of the *Pp-pme in planta*, the full-length coding sequence was amplified from a nematode cDNA library (S1 Table). RT-PCR products were cloned into the pCR TOPO II vector (ThermoFisher Scientific, MA, USA), digested with *Eco*RV restriction enzyme (restriction site was incorporated into both PCR primers), gel-purified and sub-cloned into the *Eco*RV-linearized PVX-based vector pP2C2S (obtained from D. Baulcombe, Sainsbury Laboratories, Norwich, England) [43]. The integrity of all clones was verified by automated sequencing. pP2C2S plasmids were linearized with *Spe*I, and capped transcripts were generated from cDNA clones using Ambion’s T7 mMessage Machine kit (ThermoFisher Scientific, MA, USA). The transcripts were mechanically inoculated onto fully expanded leaves of three *N*. *benthamiana* plants. Transcripts were also produced from pP2C2S plasmids without inserts (“empty” PVX vector) and inoculated onto three plants to serve as controls representing a wild-type PVX infection (PVX-WT). Three more plants were buffer-inoculated to serve as negative (“healthy”) controls. Inoculated plants were monitored daily for symptoms. The inoculation experiments that included all sets of plants of each variant was repeated three times. At 14 days after inoculation leaves and roots were photographed and collected, snap-frozen in liquid nitrogen and stored at -80°C until RNA extraction. cDNA was synthesized using the same protocol mentioned above. Semi-quantitative RT-PCR analyses were performed for transcript detection of *Pp-pme* or PVX wild-type fragments from infected *N*. *benthamiana* leaves and roots (S1 Table).

### Protein identification

Symptomatic leaves from two individual plants infected with PVX-Pp-pme recombinant virus vector and two plants infected with empty vector (PVX-WT) were sent to Bioproximity LLC (Virginia, USA) for protein extraction and identification services to reveal the top most abundant protein groups expressed in the infected plants. All procedures were performed according to the company protocols and protein assays were carried out on a Thermo Q-Exactive HF-X Orbitrap mass spectrometer (https://www.bioproximity.com/protein-identification). The obtained Ultra Performance Liquid Chromatography—Tandem Mass Spectrometer (UPLC-MS/MS) peptide datasets (mzML format for proteomics mass spectrometric data) were exported as Mascot generic format (.mgf) file using PRIDE Inspector software [44]. PeptideShaker version 1.16.36 [45] was used for protein identification and validation. Protein identification was performed against a concatenated target/decoy version of *N*. *tabacum* cv. TN90 proteome (ID UP000084051) [46] retrieved from the UniProtKB (Universal Protein Resource Knowledgebase—http://www.uniprot.org/), with 73,605 proteins complemented with Pp-PME protein sequence (3 Jan, 2019). Decoy sequences were generated by reversing the target sequences in SearchGUI 3.3.11 [47]. Identifications settings were: Trypsin with a maximum of 1 missed cleavages; 10.0 ppm for precursor m/z tolerance (MS1) and 0.5 Da for fragment m/z tolerance MS2; fixed modifications: carbamidomethylation of C (+57.021464Da), and variable modifications: deamination of N (+0.984016 Da), deamination of Q (+0.984016 Da), oxidation of M (+15.994915 Da), pyrolidone from E (-18.010565 Da) and pyrolidone from Q (-17.026549 Da). Peptides and protein inferences were identified from the spectrum of identification results. Protein Inference was considered with 100% confidence when identification was based on 2 or more validated peptides. Peptide Spectrum Matches (PSMs), peptides and proteins were validated at 1.0% False Discovery Rate (FDR) estimated using the decoy hit distribution. UPLC-MS/MS data with respective protein inference were deposited at the proteomics data repository PRIDE Archive (http://www.ebi.ac.uk/pride/archive/) with the dataset identifier PXD012419.

## Results

### *Pp-pme* genomic and cDNA coding sequences

**Fig 1:**
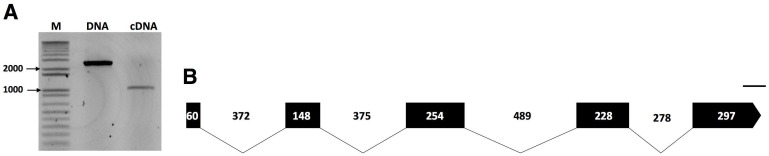
Molecular characterization of the pectin methylesterase (*Pp-pme*) of *Pratylenchus penetrans*. (A) Amplicons of both genomic (2,501 bp) and cDNA coding (987 bp) sequences of *Pp-pme*, respectively. (B) Schematic representation of the *Pp-pme* gene structure. Relative positions and respective sizes of the exons are indicated by dark boxes and introns by lines.

Data mining of the transcriptome assembly of *P*. *penetrans* revealed a transcript of 415 bp with high similarity to a pectin methylesterase gene from a bacterium *Chitinophaga* sp. CF118 (WP_090106049.1, BLAST e-value of 1,31e-111). To validate whether this transcript originated from a prokaryote or an eukaryote, BLAST search analyses were initially performed using the skimming genome assembly of *P*. *penetrans* [32], which confirmed the presence of a PME-coding gene in *P*. *penetrans*. The cloned genomic DNA amplicon revealed a transcribed genomic sequence of 2,501 bp, while the cloned cDNA amplicon contained a putative open reading frame of 987 bp for the isolate NL 10p RH (Fig 1A). The exon/intron boundaries of the genomic sequence were determined by aligning both genomic and cDNA sequences (Fig 1B and S1 Fig). The genomic sequence revealed the presence of four introns (368, 377, 493 and 282 bp long), all following the canonical GT/AG splicing site (Fig 1B). The coding sequence has an overall GC content of 56.36%, with a GC1 of 52.25%, GC2 of 45.95%, and GC3 of 70.87% (S2 Table). Using the same set of primers, the full-length cDNA sequence of a *P*. *penetrans* A44L4 isolate collected in Portugal was cloned, thus confirming the presence of a PME gene in a distant geographic isolate. The cloned *Pp-pme* cDNA sequence of the Portuguese isolate yielded a coding sequence of 981 bp and had 94.74% identity to the *Pp-pme* of the USA isolate (S2A Fig). The translated protein sequences of both isolates share 95.73% identity (S2B Fig). The cloned sequences of both isolates were deposited at NCBI as *Pp-pme* with the accession numbers MK295632 and MK295633, respectively. Unless otherwise stated, all the following analyses were conducted with *P*. *penetrans* NL 10p RH isolate.

### Characterization and sequence analysis of the predicted Pp-PME protein

**Fig 2:**
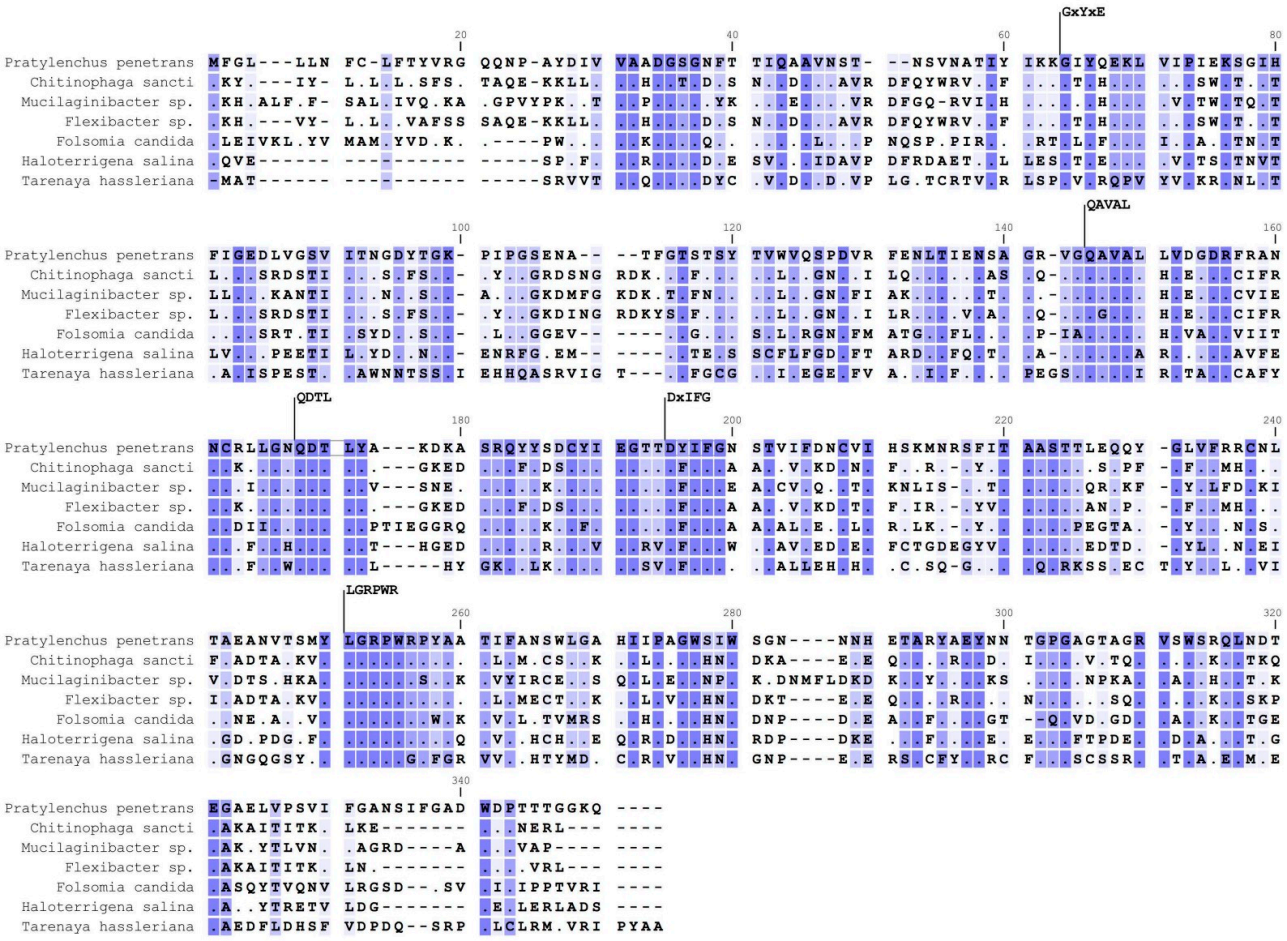
Multiple sequence alignment of the predicted Pp-PME protein of *Pratylenchus penetrans* with PMEs of other organisms. Representative species of bacteria (*Chitinophaga sancti*, *Mucilaginibacter* sp., *Flexibacter* sp.), collembolan (*Folsomia candida*), Archaea (*Haloterrigena salina*) and plants (*Tarenaya hassleriana*) were used. The five conserved sequence segments typical of PME proteins are numbered according to their positions in the corresponding Pp-PME predicted protein (64_GxYxE, 146_QAVAL, 168_QDTL, 195_DxIFG, and 251_LGRPW). Conserved residues among species are indicated by dark blue shading and dots, whereas similar residues are represented in light blue using a threshold for shading of 50% similarity. The accession numbers corresponding to each species are presented in S3 Table.

*In silico* translation of the *Pp-pme* full-length cDNA revealed a protein sequence of 328 amino acids with a predicted molecular weight of 35.815 kDA and a pI of 5.87. The protein was predicted to have an N-terminal signal peptide of 16 amino acids as determined by SignalP v. 4.0, with a cleavage site located between positions 16 and 17 (VRG-QQ), and no predicted transmembrane domain. Pfam domain search and InterPro scan confirmed this protein as a member of the CE8 family (pfam01095; InterPro IPR000070). PMEs can also be distinguished on the basis of specific signature patterns in their amino acid sequences [48]. Based on the multiple sequence alignment of *Pp-PME* with PMEs of other organisms, the following five conserved sequence segments typical of PMEs were identified: 64_GxYxE, 146_QAVAL, 168_QDTLY, 195_DxIFG and 251_LGRPR (Fig 2).

**Fig 3:**
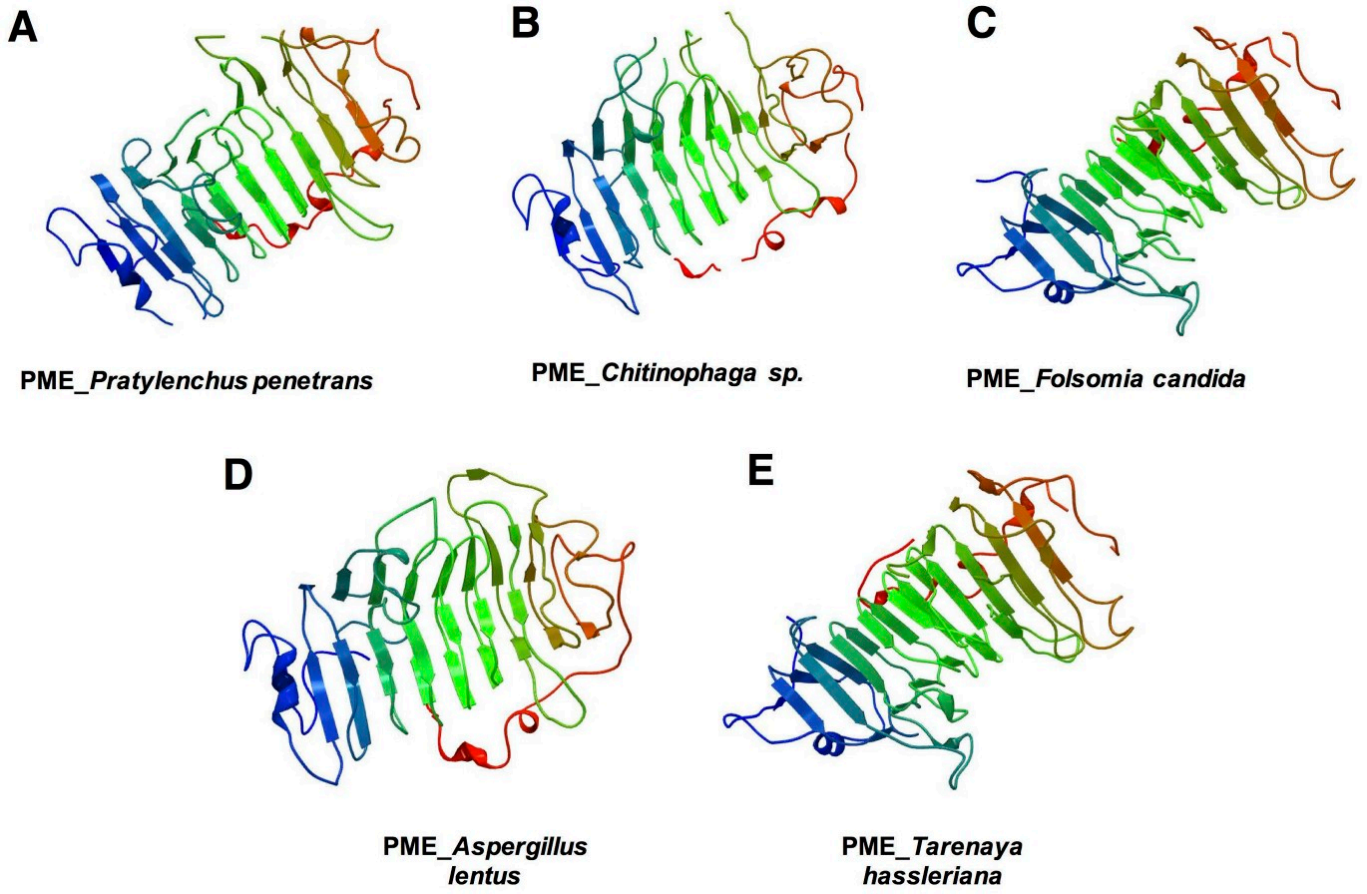
Three-dimensional model predicted for the pectin methylesterase of different organisms. (A) *Pratylenchus penetrans*, (B) bacteria (*Chitinophaga* sp.), (C) collembolan (*Folsomia candida*), (D) fungi (*Aspergillus lentus*), and (E) plants (*Tarenaya hassleriana*). The models of *P*. *penetrans* and *Chitinophaga* sp. were based on the three-dimensional model of the *Erwinia chrysanthemi* (Phyre2 fold library ID: d1gq8a), *F*. *candida* and *T*. *hassleriana* were based on the model of *Daucus carota* (Phyre2 fold library ID: d1qjva), and *A*. *lentus* on the model of *A*. *niger* (Phyre2 fold library ID: c5c1cA), respectively. The N-terminal is indicated in blue and the C-terminal is shown in red.

A three-dimensional model of Pp-PME of *P*. *penetrans* (Fig 3) was predicted using the PME of *Erwinia chrysanthemi* as template (NCBI TaxId: 556), which was the top hit species obtained by a BLASTp similarity search against the Protein Data Bank. There was 41% identity and 293 residues were covered with 100% confidence. This structure displayed a single-stranded, right-handed beta-helix fold (SCOPe: 51125) composed predominantly of beta-strands (class All beta proteins, SCOP 48124). One alpha-helix was predicted at the N-terminal end of the PME (14 amino acids) and another alpha-helix (5 amino acids) was predicted and at the C-terminal end. The latter was followed by a beta-strand (5 amino acids) and a short alpha-helix (4 amino acids). For the other organisms, the model of each corresponding PME was predicted with 100% confidence (Fig 3).

### Phylogenetic analysis of Pp-PME

Although PMEs occur naturally in both prokaryotes and eukaryotes, prior to this work there was no record of a PME within the phylum Nematoda. BLAST searches were performed using the Pp-PME protein sequence as a query against different public databases. The top 15 BLAST hits, consisting of PMEs of bacteria origin only, are presented in Table 1. The amino acid sequence of Pp-PME was found to be most closely related to PMEs of different bacteria of the phylum Bacteriodetes (E-values ranging from 1e-111 to 1e-105), showing 56.86% identity with *Chitinophaga* sp. CF118, 53.65% with *Mucilaginibacter* sp. PPCGB 2223, and 53.54% with *Flexibacter* sp. ATCC35208. When BLASTp searches were performed against other organisms for which PMEs have been previously identified (i.e. archaea, fungi, insects, and plants) (S3 Table), the highest homology of the Pp-PME was found with the PME of the soil arthropod *Folsomia candida* (Arthropoda: Collembola: Isotomidae) (51.58% protein identity; E-value = 7e-96), and with the PME of *Haloterrigena salina* (Halobacteriaceae: Euryarchaeota: Archaea) (46.18% protein identity; E-value = 4e-83). BLAST results against insects and plants showed a lower identity (up to 43.17% and 40.62%, respectively) against the nematode Pp-PME (S3 Table). Despite exhaustive BLAST searches against the large number of sequences and genomes available for other RLNs or PPNs, no homologues were found in any available nematode sequence datasets. The analysis of codon usage of Pp-PME and the corresponding top hit of each group of organisms (S4 Table) indicates that Pp-PME exhibits codon usage that is more similar to other eukaryotic species, than to prokaryotes.

**Table 1:** List of top BLAST hit sequences obtained by BLASTp analyses (E-value <1e-5) using *P*. *penetrans PME* as query.

Species	Accession no.	Organism (Phylum: Family)	BLASTp (E-value)	Identity (%)
***Chitinophaga* sp. CF118**	WP_090106049.1	Bacteriodetes: Chitinophagaceae	1,31e-111	56.86
***Chitinophaga* sp. YR627**	WP_089809083.1	Bacteriodetes: Chitinophagaceae	8,21e-110	54.69
***Chitinophaga sancti***	WP_072360114.1	Bacteriodetes: Chitinophagaceae	9,39e-113	54.63
***Chitinophaga filiformis***	WP_089838976.1	Bacteriodetes: Chitinophagaceae	1,06e-107	54.37
***Chitinophaga* sp. YR573**	WP_089782777.1	Bacteriodetes: Chitinophagaceae	2,05e-111	53.99
***Chitinophaga pinensis***	WP_012789484.1	Bacteriodetes: Chitinophagaceae	9,27e-111	53.99
***Mucilaginibacter* sp. PPCGB 2223**	WP_066007482.1	Bacteriodetes: Shingobacteriaceae	2,12e-105	53.65
***Flexibacter* sp. ATCC 35208**	WP_083721311.1	Bacteriodetes: Cytophagaceae	2,28e-109	53.54
***Mucilaginibacter paludis***	WP_008506893.1	Bacteriodetes: Shingobacteriaceae	5,15e-109	53.50
***Mucilaginibacter* sp. OK268**	WP_090463524.1	Bacteriodetes: Shingobacteriaceae	1,50e-105	53.07
***Spirosoma aerolatum***	WP_080054327.1	Bacteriodetes: Cytophagaceae	2,23e-106	52.98
***Pedobacter ginsenosidimutans* KACC14530**	KRT18230.1	Bacteriodetes: Shingobacteriaceae	1,55e-106	52.12
***Pedobacter ginsenosidimutans***	WP_083505239.1	Bacteriodetes: Shingobacteriaceae	2,28e-106	52.12
***Pedobacter* sp. PACM 27299**	WP_062548895.1	Bacteriodetes: Shingobacteriaceae	6,99e-107	51.09
***Rufibacter roseus***	WP_066624157.1	Bacteroidetes: Hymenobacteraceae	2,19e-105	50.61

**Fig 4:**
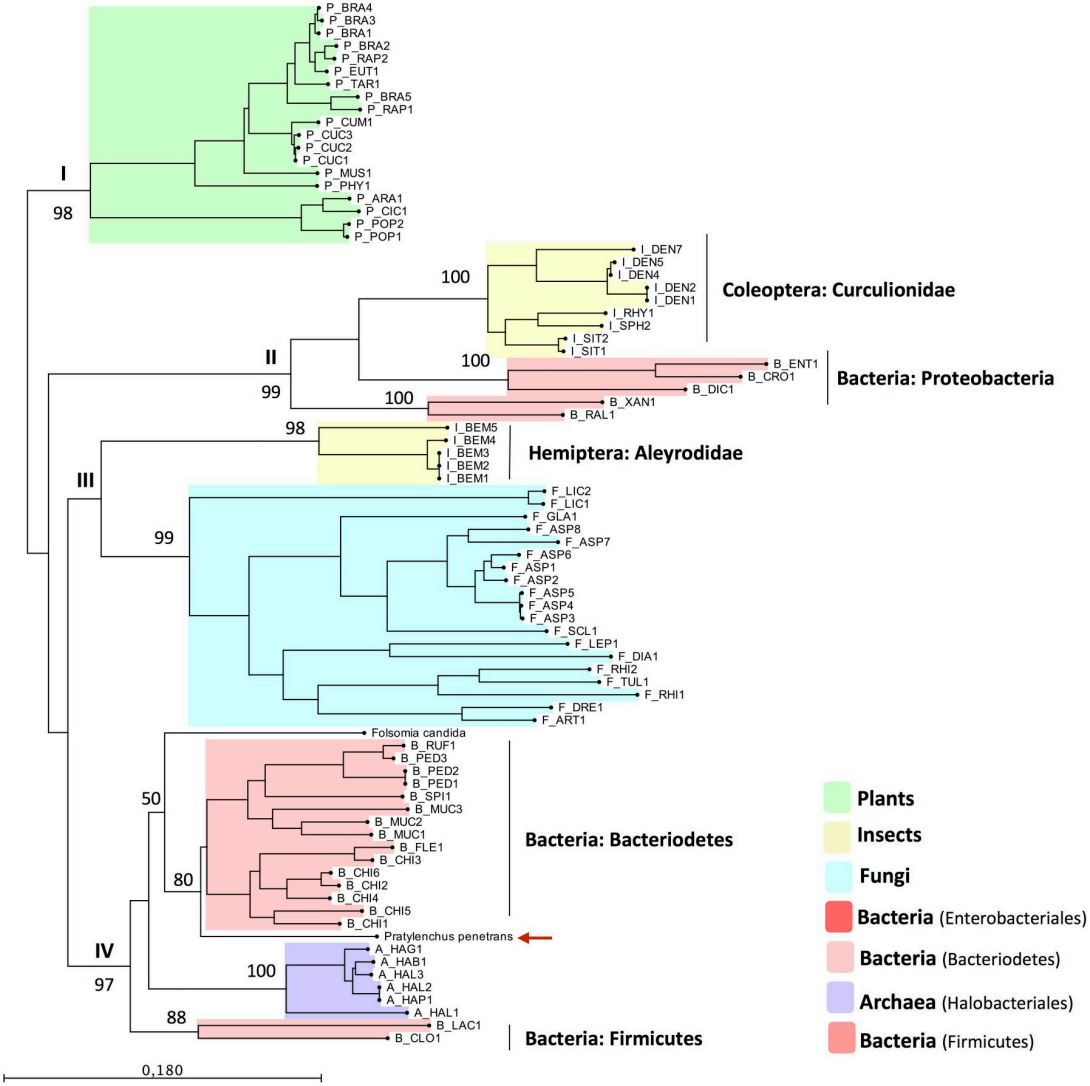
Phylogenetic tree based on the closest homology to the catalytic domain of Pp-PME. The PME sequences across different *taxa* were chosen based on the top BLAST hits against the Pp-PME predicted pectinesterase domain. The corresponding species names and range of e-values are presented in S3 Table. The red arrow indicates the position of *P*. *penetrans* PME. The phylogenetic tree was deduced by Maximum Likelihood with the “Whelan and Goldman” (WAG) model with discrete gamma distribution and 1000 bootstrap replicates.

The predicted pectinesterase domain sequence (pfam01095, position 23–307 aa, E-value = 1.8e-68) of Pp-PME was then aligned with the corresponding domain sequences of the top hit PME proteins of 53 eukaryotic species, including those of fungi, insects, and plants, as well as 29 prokaryotic species distributed among bacteria and Archaea (S3 Table). In order to infer the phylogenetic relationship of the Pp-PME and other organisms, ML analyses were performed (Fig 4). These analyses revealed a clear separation of the PMEs of plant origin with the remaining PMEs (i.e. archaea, bacteria, fungi, insects, and *P*. *penetrans*), and highly supported by our bootstrap results (>98%). The PMEs of the remaining *taxa* could be separated into three main phylogenetic clades, which is also highly supported by the bootstrap analyses. Interestingly, the PMEs of the insects of the family Curculionidae (Coleoptera) clustered together with bacterial PMEs of the Proteobacteria phylum, while the PMEs of the whitefly *B*. *tabaci* (family Aleyrodidae, Hemiptera) grouped with a monophyletic clade formed by fungi only. As denoted by the protein alignment described above, Pp-PME clustered with bacterial sequences of the phylum Bacteriodetes (80% bootstrap value), including the soil collembolan *F*. *candida* (family Isotomidae, Collembola), although with a lower bootstrap value. All remaining PME sequences of bacteria (Phylum Firmicutes) and Archaea formed well-separated sub-clusters.

### Expression pattern profile of *Pp-pme* at different stages of nematode development

**Fig 5:**
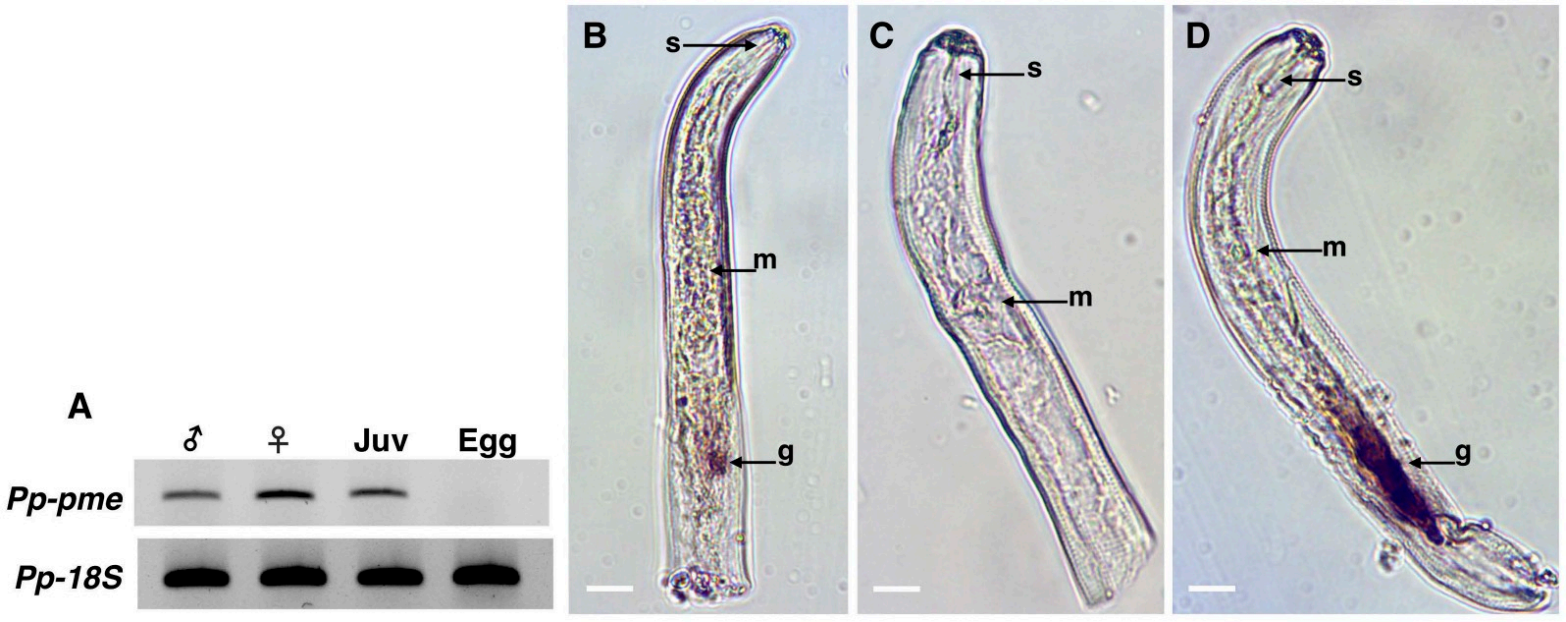
Expression and localization of *Pratylenchus penetrans Pp-pme* transcripts. (A) Determination of *Pp-pme* expression in different nematode developmental stages of *P*. *penetrans* by semi-quantitative RT-PCR. As a positive control, all cDNA templates were amplified with primers derived from the *18S* gene of *P*. *penetrans*. The nematode stages were separated as males, females, juveniles (J2-J4), and eggs. (B-C) Detection of the *Pp-pme* transcripts by *in situ* hybridization. Nematode sections were hybridized with antisense (B), or sense (C) *Pp-pme* digoxigenin-labeled cDNA probes. (D) As a positive control, *in situ* hybridization was performed with the antisense probe designed for the CWDE (*Pp-eng-1*) specifically localized within the esophageal glands g: esophageal glands; m: metacorpus; s: stylet.

The expression pattern of *Pp-pme* was determined by semi-quantitative RT-PCR for different nematode developmental stages [eggs, juveniles (J2-J4), adult males and females] (Fig 5A). *Pp-pme* expression was only detected for nematode motile stages, whereas no expression was detected from the eggs. Amplification of *18S* rDNA gene fragment was used as positive control in this experiment.

Identification of the gene-specific transcripts within the nematode tissues can provide insights into putative functions of the *Pp-pme*. Therefore, we performed whole mount *in situ* hybridization for detection of transcripts within the nematode tissues. As determined by the antisense probe, the *Pp-pme* transcripts accumulated within the esophageal glands of the nematode (Fig 5B), while no signal was detected using the sense probe of the *Pp-pme* as control (Fig 5C). As a positive control, *in situ* hybridization was performed with the antisense probe designed for a CWDE (*Pp-eng-1*) that specifically localizes within the esophageal glands (Fig 5D). The localization of the *Pp-pme* within the glands and the presence of a N-terminal signal peptide of the deduced Pp-PME protein suggest its secretion by the nematode into the host.

### *Pp-pme* transcript expression in host roots after nematode infection

**Fig 6:**
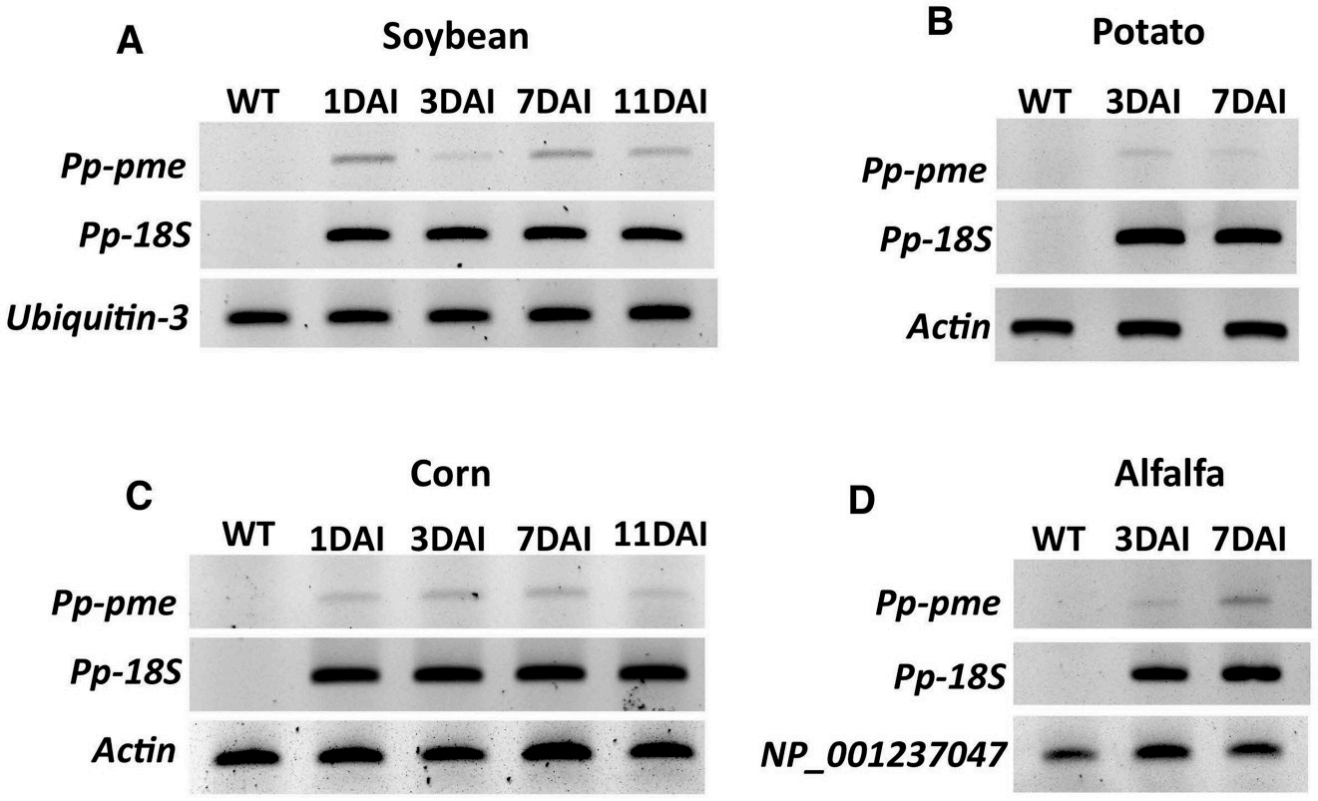
Semi-quantitative RT-PCR showing the transcript levels of *Pp-pme* in different host plants at different time points after infection. Total RNA extracted from nematode infected roots of economically important host plants (A) soybean, (B) potato, (C) corn, and (D) alfalfa was used to validate the relative expression of *Pp-pme* at different days after nematode infection (DAI). The nematode *18S* rDNA gene was used as internal control to validate the presence of *P*. *penetrans* within the infected roots, while specific plant reference genes were used for each specific host plant. Wild-type (WT) correspond to non-infected plants.

The expression of the *Pp-pme* was analyzed during interaction with different host plants (e.g. roots of alfalfa, corn, potato and soybean) by semi-quantitative RT-PCR at early time points after nematode infection (Fig 6). In each respective nematode-plant interaction the expression of the *Pp-pme* could be detected, showing that this gene is actively transcribed during the nematode’s interaction with the host plants.

### Transient expression of *Pp-pme* in *Nicotiana benthamiana*

**Fig 7:**
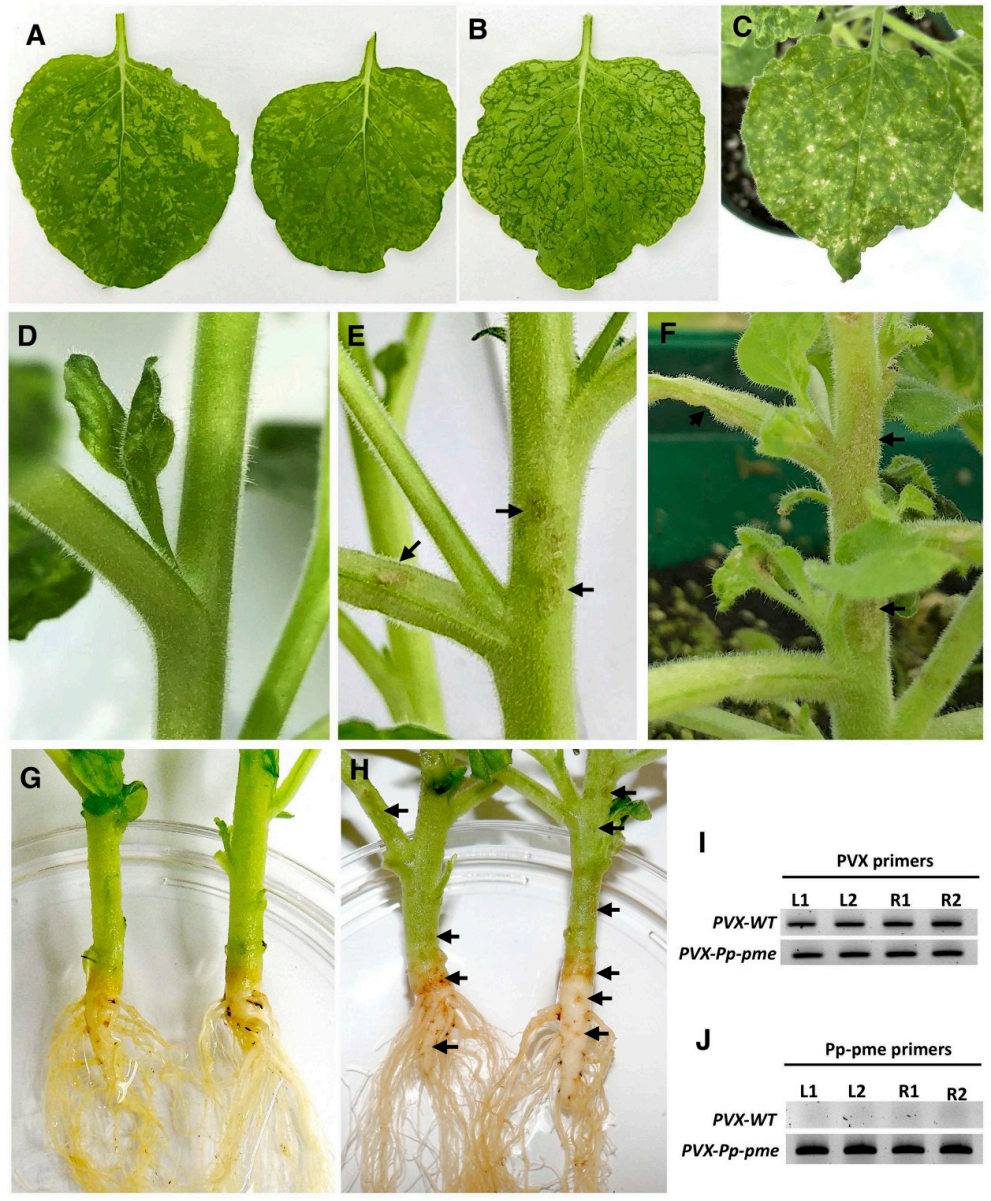
Phenotypic changes in *Nicotiana benthamiana* plants infected with the recombinant *PVX-Pp-pme* virus. All photos were taken 14 days after inoculation. (A) characteristic mosaic-like symptom developed in *N*. *benthamiana* plants infected with transcripts generated from the empty PVX vector. (**B-C**) Distinct leaf chlorosis symptoms (B) and lesion-like spots (C) developed in *N*. *benthamiana* plants infected with *PVX-Pp-pme* transcripts, respectively. (D) Stem and branches of the *N*. *benthamiana* plant infected with transcripts generated from the empty PVX vector displayed no symptoms. (E-F) Lesion-like symptoms observed on stems and branches of *N*. *benthamiana* plants infected with *PVX-Pp-pme* transcripts. (G) Stem and roots of *N*. *benthamiana* plant infected with empty PVX vector displayed no symptoms. (H) Browning in the area of the stem-root joint and lesion-like spots observed on the roots of *N*. *benthamiana* plants infected with *PVX-Pp-pme* transcripts. (I-J) Detection of PVX-WT and *PVX-Pp-pme* transcripts in inoculated *N*. *benthamiana* plants by semi-quantitative RT-PCR. L1, L2: leaves from two independently-inoculated plants; R1, R2: roots from two independently-inoculated plants.

Plant-parasitic nematodes are able to secrete effector proteins directly into the apoplasm or cytoplasm of the plant cell. CWDEs are often secreted by PPNs to the apoplasm during invasion of the plant roots [49]. These CWDEs are thought to be involved in cell wall disassembly thus facilitating the penetration and migration process of the nematode into the roots. This is indicative that the Pp-PME could be also secreted into the apoplast by the nematode and be involved in the invasion of the roots. To direct the nematode PME into the secretory pathway of the host cell, and to assess putative *Pp-pme* functional role, the full-length coding sequence of the *Pp-pme* gene, including the signal peptide, was transiently expressed in *N*. *benthamiana* using a PVX-based vector. Phenotypic differences between symptoms generated by the recombinant virus containing *Pp-pme* and by the empty PVX vector are shown in Fig 7. Systemic expression of the *Pp-pme* gene via the PVX vector in *N*. *benthamiana* resulted in more pronounced chlorosis, vein clearing and yellowing of the leaves starting 10–14 days after inoculation (Fig 7B). In some cases, lesion-like symptoms were observed on the leaves of PVX-*Pp-pme*-infected plants (Fig 7C). Plants expressing empty PVX transcripts displayed characteristic mosaic-like symptoms (Fig 7A). In addition, all plants inoculated with *PVX-Pp-pme* exhibited distinct lesion-like symptoms in different areas of the stem, branches (Fig 7E and 7F) and main root (Fig 7H), which were absent in plants infected with empty PVX vector (Fig 7D and 7G). Semi-quantitative RT-PCR conducted confirmed expression of the *Pp-pme* gene in plants inoculated with *PVX-Pp-pme* (Fig 7J), while a fragment of PVX plasmid confirmed the expression of PVX transcripts in inoculated plants (Fig 7I).

To detect the possible accumulation of the Pp-PME in the leaves of *N*. *benthamiana* plants, UPLC-MS/MS analyses were performed with proteins extracted from the leaves of PVX-Pp-pme and PVX-WT plants. The peptides identified by mass spectrometry were matched to a set of 73,605 proteins sequences of *N*. *tabacum*, and complemented with the predicted Pp-PME protein sequence (S6A and S6B Table). In both PVX-Pp-pme plants, seven to nine peptides were identified that were 100% identical to Pp-PME (Table 2 and S3 Fig). No Pp-PME-related peptides were retrieved from two PVX-WT plants (S6C and S6D Table).

**Table 2:** List of Pp-PME peptides identified in the peptide datasets generated by UPLC-MS/MS from leaves expressing the PVX-Pp-PME construct.

Library/Peptide #	Peptide Sequence	Peptide Start	Peptide End	Score (%)
Library 1				
1	SFITAASTTLEQQYGLVFR	206	225	100
2	VGQAVALLVDGDRFR	136	151	100
3	FENLTIENSAGR	124	136	100
4	GQAVALLVDGDRFR	137	151	100
5	LLGNQDTLYAK	156	167	100
6	RSFITAASTTLEQQYGLVFR	205	225	100
7	SFITAASTTLEQQYGLVFRR	206	226	100
8	SGIHFIGEDLVGSVITNGDYTGK	75	98	100
9	VGQAVALLVDGDR	136	149	100
Library 2				
1	FENLTIENSAGR	124	136	100
2	LLGNQDTLYAK	156	167	100
3	SFITAASTTLEQQYGLVFR	206	225	100
4	SFITAASTTLEQQYGLVFRR	206	226	100
5	SFITAASTTLEQQYGLVFR	206	225	100
6	VGQAVALLVDGDR	136	149	100
7	VGQAVALLVDGDRFR	136	151	100

## Discussion

In this work, we report the identification, characterization, and phylogenetic status of a putative pectin methylesterase gene PME that encodes a cell wall-degrading enzyme in the RLN, *P*. *penetrans*. Several previous studies have shown that PPNs possess a set of CWDEs to degrade the plant cell wall and thus facilitate their invasion and migration through the plant host tissues. The diversity and number of CWDEs endogenous to each PPN do not only reflect the complex nature of the plant cell wall and its structural components, but also indicate an elaborate parasitic strategy and adaptation of these obligatory plant-pathogens [50]. Although different families of CWDEs have been reported in a wide range of PPNs, including RLNs, prior to this work, no pectin methylesterase gene has been identified in the phylum Nematoda.

Although bacteria (e.g. *Wolbachia*) have been reported from within different parts of the *P*. *penetrans* body [51], the features of the *Pp-pme* gene exclude the possibility of a prokaryotic contamination. The gene structure of this new nematode CWDE encompasses typical eukaryotic features with four characteristic spliceosomal introns (GC/AG splice sites). The presence of a putative signal peptide in the corresponding Pp-PME sequence, coupled with its transcript expression in the esophageal glands of the nematode, highlights its potential importance in the parasitic process of *P*. *penetrans*. This is in agreement with our previous observations of other CWDEs found in this particular species [31], which were localized in the esophageal glands of the nematodes and potentially secreted into the host tissues.

Despite all PMEs being classified under the same family, their mode of action is likely different, depending on the pH and ionic environment, substrate specificity, and origin [17]. In plants, PMEs are encoded by a large family of genes, which emphasizes their functional diversity within the plant tissues [9]. In bacterial and fungal pathogens, PMEs play a critical role in their virulence [52], since their secretion has been related to enzymatic degradation of the pectin polysaccharides of the plant cell walls. *P*. *penetrans* has a wide range of host plants and is capable to parasitize on both mono- and dicotyledonous plants. The components of the cell wall in monocots and dicots vary extensively, thus requiring some plasticity in the substrate specificity of the enzymes secreted by the nematode. Our results indicate that *Pp-pme* is expressed during the early infection time points in different host plants. This is in line with the expression of other CWDEs identified in *P*. *penetrans*, such as pectin lyases and different glycoside hydrolase (GH) gene families [31]. Degradation of the plant cell wall pectin network requires the synergistic action of different pectolytic enzymes, such as polygalacturonases (GH28), PLs, and PMEs. PME catalyze the de-methylesterification of pectin, a major component of plant cell wall, where their activity is finely tuned through endogenous inhibitors. Plant-derived PMEs can contribute toward immunity against pathogens [53], while pathogenic PME may function to promote disease [18, 19]. The activity of PMEs can improve cell wall accessibility for other CWDEs and consequently accelerate cell wall degradation [9, 10, 11]. The correctness of the discovered *Pp-pme* genetic sequence was confirmed by its transient expression *in planta*, where it translated into a PME-like protein. Therefore, it is plausible that the synchronized expression of the nematode PME with other CWDEs during infection could induce changes in the properties of the plant cell wall, contributing to penetration and migratory activity of the nematode. Speculatively, the symptoms observed on PVX-Pp-pme infected plants could be potentially related to the activation of the plant defense reactions against this effector-like protein. Although, further experiments are needed to confirm this.

To date, PMEs are restricted to plants, bacteria, and fungi and are exclusively found in a small number of insect species belonging to the family Curculionidae [23, 26, 54], the whitefly and the springtail [24, 25]. The predicted pectinesterase amino acid domain of the Pp-PME showed significant similarity to different bacterial PMEs. Phylogenetic analyses performed in this work, indicates that this gene has most likely been acquired by horizontal gene transfer (HGT) into *P*. *penetrans*. Horizontal gene transfer events of other CWDEs from ancestral microbial donors into sedentary and migratory PPNs of the Tylenchida have been extensively reported [50, 55]. One common characteristic of these CWDE genes, which was also observed for the genomic *Pp-pme* sequence, is the presence of long introns [56, 57], in contrast to the average intron size reported for the available nematode genomes [58]. The functions of the transferred gene products are often linked to particular steps of nematode-plant interaction, particularly to the parasitism of PPNs. As mentioned above, *Wolbachia* endosymbionts have been found in different tissues of *P*. *penetrans* [51], which could be seen as a potential route for HGT of the transmissible genomic fragments to the nematode. However, the symbiotic bacteria described so far from nematodes, including the most recently characterized *Wolbachia* strain (wPpe) isolated from *P*. *penetrans* [46], are not known to encode any of the CWDEs that have been potentially acquired by HGT [50].

The PME sequences of the bacterial origin used in this study are highly heterogeneous and were placed by the phylogenetic analysis into different branches of the phylogenetic tree correlating with different niches and *taxa*. While PME sequences of flying insects of the family Curculionidae strongly correlate with Proteobacteria, the whitefly *B*. *tabaci* clustered together with PME sequences of the fungal origin. The diversity of the genes encoding PME in the different groups of insects, support the idea that these PMEs have potentially been acquired multiple times independently by their receiver taxa [23]. In many cases, genes encoding CWDEs in PPNs seemed to be most closely related to sequences found in bacteria that inhabit the soil [55]. The predominant putative ancestral donors found so far are related to bacteria of the Phyla Proteobacteria, Actinobacteria, Firmicutes, and Bacteriodetes [55, 59]. In line with these findings, *P*. *penetrans* PME is most similar to PMEs of bacterial sequences of the phyla Bacteriodetes (Chitinophagaceae, Chitinophaga), followed by the PME sequences of the soil collembolan *F*. *candida* (Collembola: Isotomidae). Species belonging to the Bacteriodetes are specialized in the degradation of complex organic matter [60, 61], particularly the family Chitinophagaceae that showed a high ability for degradation of plant-derived carbohydrates (e.g. cellulose and chitin) [62, 63]. Remarkably, this group of bacteria has been recently reported to be a part of the rhizosphere microbiome associated with the presence of *P*. *penetrans* [63] and also found in the microbiome of *F*. *candida* [64, 65]. Similarly, the genome of *F*. *candida*, one of the most abundant arthropods in soil, showed an extensive number of carbohydrate-active enzymes potentially acquired by the HGT from soil bacteria. These enzymes enable *F*. *candida* to scavenge decaying plant matter as a food source [25]. As RLN are composed of a vast number of species with all of them occurring in the soil, it will be interesting to investigate whether they also carry a PME-coding gene.

## Supporting information

S1 Table List of primers used in this study. (XLSX)

S2 Table Percentage of GC content (GC1, GC2, GC3) of selected PMEs characterized in this study. Pp, *Pratylenchus penetrans*; Bacteria, B; Archaea, A; Fungi, F; Insect, I; Plant, P.
(XLSX)

S3 Table List of PME sequences, and respective species, used for phylogenetic analysis of Pp-PME. These sequences of each particular taxa correspond to the top BLAST hits against *P*. *penetrans* PME sequence.
(XLSX)

S4 Table Codon usage of PMEs of representative species of different *taxa*. The preferred codons are highlighted in red.
(XLSX)

S5 Table Similarity matrix among the different PME sequences obtained for different groups of organisms including the PME sequence of *Pratylenchus penetrans*. (XLSX)

S6 Table List of peptides and corresponding protein identification generated through mass spectrometry analyses. (XLSX)

S1 Fig Alignment of genomic DNA and cDNA *Pp-pme* sequences of *Pratylenchus penetrans* isolate NL10p RH. Exons and introns are indicated by green and red arrows, respectively.
(PDF)

S2 Fig Nucleotide (A) and predicted protein (B) alignments of the PME cloned from two different geographic isolates of *Pratylenchus penetrans*. Pp-PME US isolate corresponds to the isolate NL 10p RH collected in Beltsville (Maryland, US), Pp-PME PT isolate corresponds to the isolate A44L4 collected from potato fields in Portugal (Coimbra, Portugal).
(PDF)

S3 Fig Mass spectrometry (UPL-MS/MS) spectrum of the Pp-PME peptides identified in plants expressing the *PXV-Pp-pme* construct. (A) and (B) correspond to the different Pp-PME peptides identified from two protein libraries extracted from the leaves of *Nicotiana benthamiana* plants inoculated with *PVX-Pp-pme*.
(PDF)
