# Correction: Identification and characterization of the first pectin methylesterase gene discovered in the root lesion nematode *Pratylenchus penetrans*

**DOI:** 10.1371/journal.pone.0214185

**Published:** 2019-03-18

**Authors:** The PLOS ONE Staff

In the Funding statement, the FCT (Foundation for Science and Technology) project name is incorrect. The correct Funding statement is as follows: This work was supported by the FCT (Foundation for Science and Technology) postdoctoral fellowship SFRH/BPD/116030/2016 (to CSLV); the national project PTDC/AGR-PRO/2589/2014 –PratyTech (to MM), Biotechnology approaches towards control of the root lesion nematode Pratylenchus penetrans and by National Funds through FCT under the Project UID/AGR/00115/2019 (to CSLV and MM).

The publisher apologizes for this error.
