# Supplementary material for: Identification and characterization of the first pectin methylesterase gene discovered in the root lesion nematode Pratylenchus penetrans
Source: PLoS One. 2019 Feb 22;14(2):e0212540. doi: 10.1371/journal.pone.0212540 (PMC6386239; doi:10.1371/journal.pone.0212540)
Supplement: S1 Fig — Exons and introns are indicated by green and red arrows, respectively. (PDF) [file pone.0212540.s007.pdf]

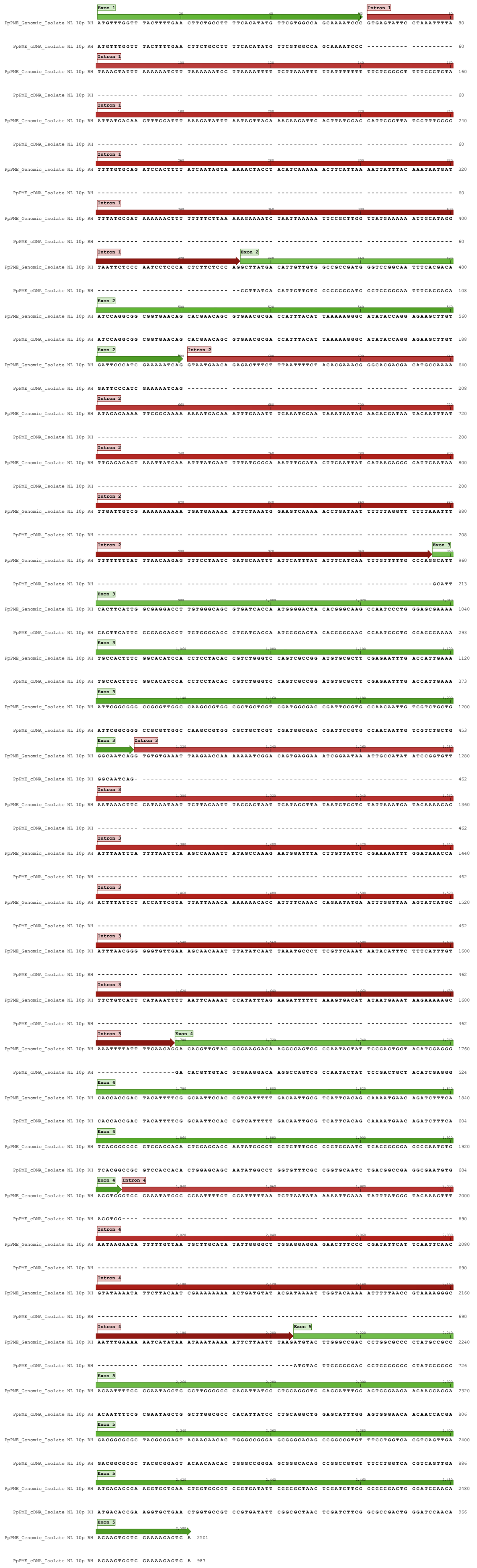

S1 Fig. Alignment of genomic DNA and cDNA *Pp-pme* sequences of *Pratylenchus penetrans* isolate NL10p RH. Exons and introns are indicated by green and red arrows, respectively.
