# Supplementary material for: Identification and characterization of the first pectin methylesterase gene discovered in the root lesion nematode Pratylenchus penetrans
Source: PLoS One. 2019 Feb 22;14(2):e0212540. doi: 10.1371/journal.pone.0212540 (PMC6386239; doi:10.1371/journal.pone.0212540)
Supplement: S2 Fig — Pp-PME US isolate corresponds to the isolate NL 10p RH collected in Beltsville (Maryland, US), Pp-PME PT isolate corresponds to the isolate A44L4 collected from potato fields in Portugal (Coimbra, Portugal). (PDF) [file pone.0212540.s008.pdf]

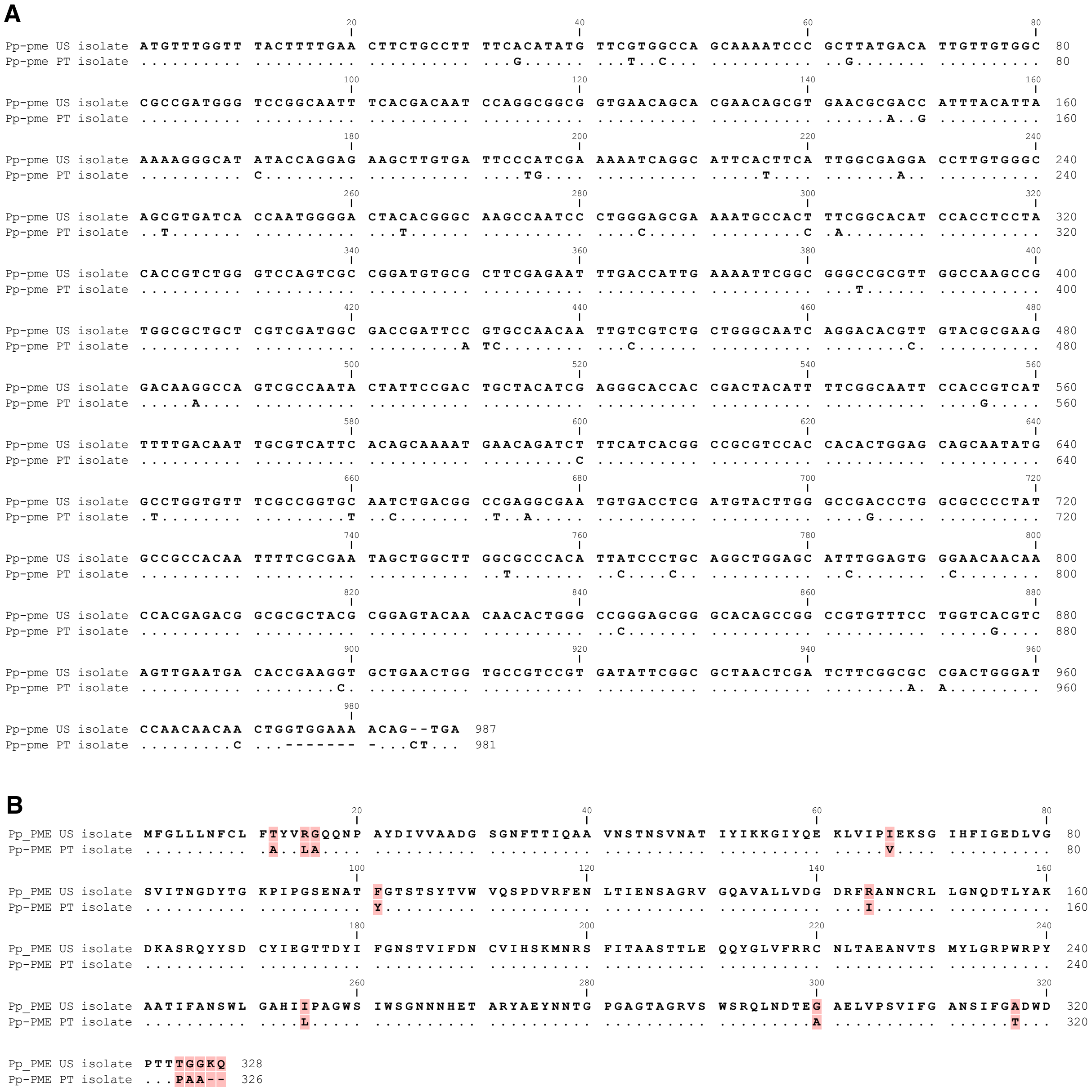

S2 Fig. Nucleotide (A) and predicted protein (B) alignments of the PME cloned from two different geographic isolates of *Pratylenchus penetrans.* Pp-PME US isolate corresponds to the isolate NL 10p RH collected in Beltsville (Maryland, US), Pp-PME PT isolate corresponds to the isolate A44L4 collected from potato fields in Portugal (Coimbra, Portugal).
