# Supplementary material for: Identification and characterization of the first pectin methylesterase gene discovered in the root lesion nematode Pratylenchus penetrans
Source: PLoS One. 2019 Feb 22;14(2):e0212540. doi: 10.1371/journal.pone.0212540 (PMC6386239; doi:10.1371/journal.pone.0212540)
Supplement: S3 Fig — (A) and (B) correspond to the different Pp-PME peptides identified from two protein libraries extracted from the leaves of Nicotiana benthamiana plants inoculated with PVX-Pp-pme. (PDF) [file pone.0212540.s009.pdf]

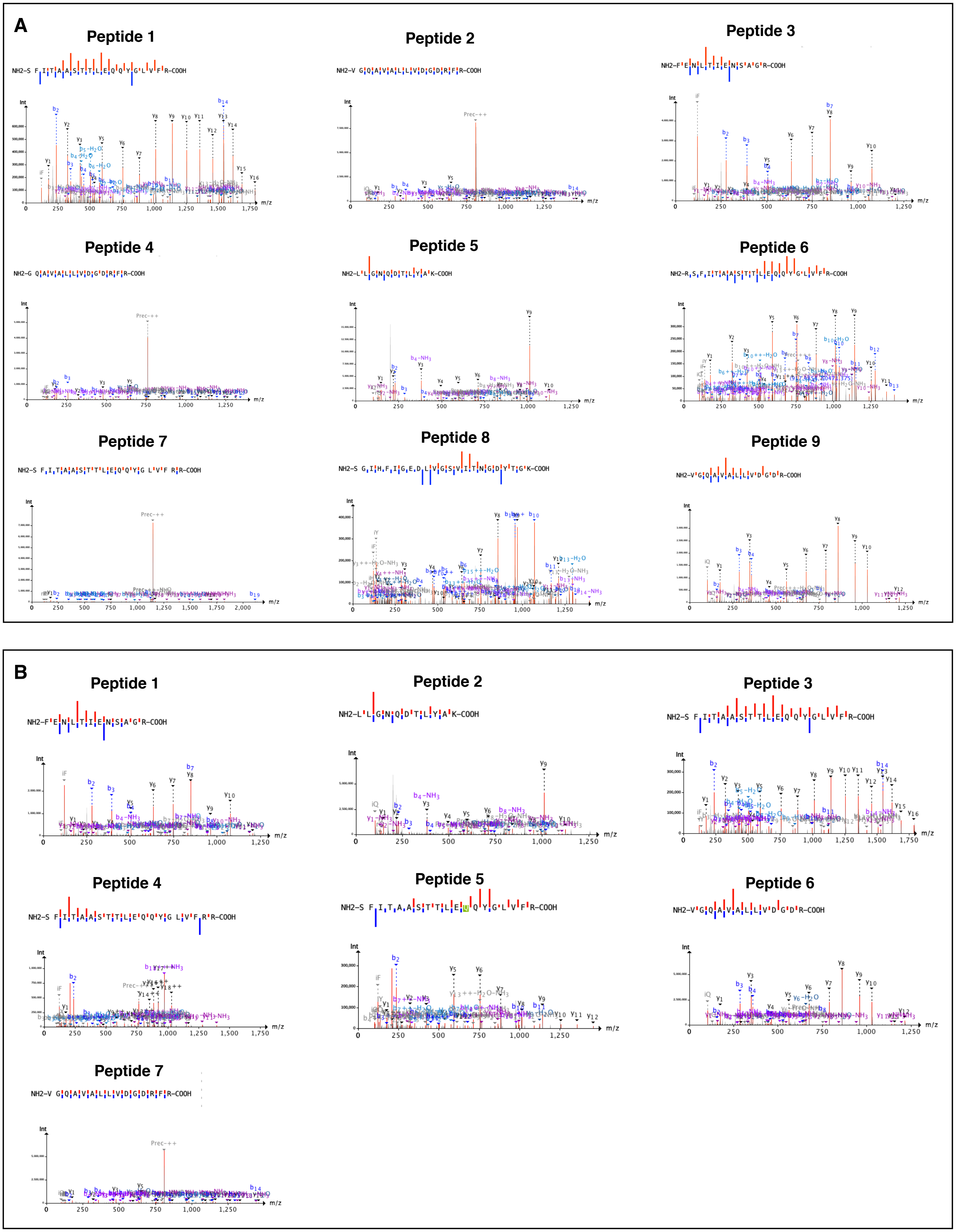

S3 Fig. Mass spectrometry (UPL-MS/MS) spectrum of the Pp-PME peptides identified in plants expressing the *PXV-Pp-pme* construct. (A) and (B) correspond to the different Pp-PME peptides identified from two protein libraries extracted from the leaves of *Nicotiana benthamiana* plants inoculated with *PVX-Pp-pme*.
